# Common Methods for Performing Mendelian Randomization

**DOI:** 10.3389/fcvm.2018.00051

**Published:** 2018-05-28

**Authors:** Alexander Teumer

**Affiliations:** ^1^Institute for Community Medicine, University Medicine Greifswald, Greifswald, Germany; ^2^Partner Site Greifswald, Deutsches Zentrum für Herz-Kreislaufforschung (DZHK), Greifswald, Germany

**Keywords:** mendelian randomization, causal inference, GWAS, bias, statistical methods

## Abstract

Mendelian randomization (MR) is a framework for assessing causal inference using cross-sectional data in combination with genetic information. This paper summarizes statistical methods commonly applied and strait forward to use for conducting MR analyses including those taking advantage of the rich dataset of SNP-trait associations that were revealed in the last decade through large-scale genome-wide association studies. Using these data, powerful MR studies are possible. However, the causal estimate may be biased in case the assumptions of MR are violated. The source and the type of this bias are described while providing a summary of the mathematical formulas that should help estimating the magnitude and direction of the potential bias depending on the specific research setting. Finally, methods for relaxing the assumptions and for conducting sensitivity analyses are discussed. Future researches in the field of MR include the assessment of non-linear causal effects, and automatic detection of invalid instruments.

## Introduction

Observational epidemiological studies made important contributions to our understanding of common diseases by identifying important risk factors. Although causal inference is of major interest as it builds a basis for intervention and prevention, it is difficult to perform using observational data from cross-sectional studies. Supposed causality was often revised e.g., by randomized controlled trials (RCTs) (1). Possible reasons for these contradicting findings include unobserved confounding, reverse causation and selection bias in the observational studies (2–4).

On the other hand, RCTs are often subject to long duration and ethical problems. Furthermore, confounding and selection bias is still a problem after the initiation of a RCT. This includes compliance problems or missing of follow-up information depending on treatment effect which may induce missing not at random problems.

During the last decade, huge efforts were undertaken searching for genetic risk factors underlying common traits and diseases. Genome-wide association studies (GWAS) revealed thousands of genetic associations predominantly based on single nucleotide polymorphisms (SNPs) including more than 950 related to cardiovascular diseases and measurements (by April 2018) and were made publically available (5). The effect sizes of these associations were often quite small (6–8), and thus their direct clinical relevance might be questioned. However, these genetic associations may help drawing causal inferences. This approach in which SNPs are used as instrumental variables (IVs) for specific exposures is called Mendelian randomization (MR) (9). By the Mendelian laws, alleles of SNPs segregate and are randomly inherited from parents to offspring. This principle can be seen analogously to the randomized treatment assignment in a RCT resulting in an unconfounded exposure-outcome relationship. Within an MR approach, the exposure represents a continuous or dichotomous risk factor of a disease, and the outcome is the disease or a disease-related trait. These traits may e.g., be blood pressure defining hypertension, or estimated glomerular filtration rate (eGFR) defining the status of chronic kidney disease. Using the MR approach, causality between exposure and outcome can be tested. During recent years, the number of MR studies to assess causality increased substantially which includes also the field of cardiovascular diseases and nephrology (10–14). Furthermore, MR analyses revealed causal effects of blood lipids on coronary heart disease (15) as well as of alcohol consumption on cardiovascular traits (16). However, given the number of potential genetic instruments and statistical methods available nowadays, there is potential for assessing causality of many more traits by conducting successful MR analyses. Nevertheless, some important assumptions have to be fulfilled to be able to estimate an unconfounded and unbiased exposure-outcome relationship thus allowing drawing causal inference. This review describes the assumptions of MR and potential biases caused by violation of these assumptions, and provides an overview of commonly applied statistical methods for conducting MR analyses using individual level data as well as using GWAS meta-analyses results.

### Estimation of the Causal Effect

**Figure 1:**
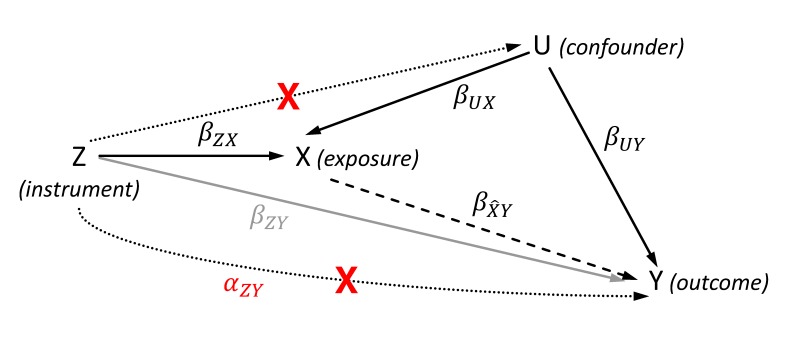
Directed acyclic graph showing the effects βof the genetic instrument *Z*, the exposure *X*, the outcome *Y* and the (unobserved) confounder *U* for illustrating the Mendelian randomization (MR). The dashed line represents the estimated causal effect $\beta_{\hat{X}Y}$ using the instrumented exposure. The dotted lines show violations of the MR assumptions 2 (lower line) and 3 (upper line), and are marked by a red cross. The $\alpha_{ZY}$ represents the effect of the instrument that affects the outcome not via the exposure in case of violating the exclusion restriction assumption. In contrast to $\alpha_{ZY}$, the gray line illustrates the SNP-outcome association with its effect $\beta_{ZY}$ that is used to calculate the two-sample MR given a valid instrument.

The general aim of the MR approach is the estimation of a causal effect of an exposure *X* on an outcome *Y* using (one or more) genetic instruments *Z* for *X* (Figure 1). Basically, the causal effect will be obtained by two sequential steps. First, the exposure is estimated from its instruments. By using valid instruments, the estimated exposure will be independent of any confounders. In the second step, the outcome is regressed on this estimated exposure thus obtaining an unconfounded and therefore causal effect estimate. The instrument *Z* is usually coded by 0, 1 and 2 per individual according to its number of coding (e.g., exposure increasing) alleles.

### 2-Stage Least Squares Estimator

Given a continuous outcome *Y* and assuming linear effects between *X* and *Y* without interaction, the causal estimate of the exposure *X* on *Y* can be estimated through a 2-stage least squares (2SLS) regression. This method performs both steps described before implicitly. In the first step, the exposure $\hat{X}$ which is independent of the confounders is estimated via the genotypes of the instruments by calculating the fitted values from the regression of *X* on *Z*. In the second step, the causal effect estimate $\beta_{\hat{X}Y}$ is obtained by regressing *Y* on $\hat{X}$. As both steps are performed in a single model instead of two separate regressions, the variation of both *Z* and $\hat{X}$ is taken into account which is required for obtaining correct standard errors (SE) of $\beta_{\hat{X}Y}$ (17). The 2SLS regression can be calculated by standard methods in statistical software packages like R (18) using the function *tsls* of the package SEM, or by the STATA software (https://www.stata.com/) using the command *ivregress*. The 2SLS was included in an MR of testosterone with cardiometabolic risk factors, but the single study analysis limited the statistical power substantially (19).

### Ratio Estimator

Alternatively, the causal effect can be estimated by triangulation without the need of calculating $\beta_{\hat{X}Y}$ from the exposure-outcome association directly. The principle of this method is illustrated through Figure 1: the standard approach (including 2SLS) for obtaining the causal effect $\beta_{\hat{X}Y}$ follows the path from the instrument *Z* via *X* to *Y*. In this case, the direct effect $\beta_{ZY}$ of the instrument on the outcome *Y* equals the product of effects underlying the path mediated by the exposure, i.e.,$\beta_{ZY}=\beta_{ZX\;}\cdot \beta_{\hat{X}Y}$. By rearranging this equation, the causal effect can be estimated through dividing the effect of the IV on the outcome ($\beta_{ZY}$) by the effect of the IV on the exposure ($\beta_{ZX}$):$\beta_{\hat{X}Y}=\beta_{ZY}/\beta_{ZX\;}$. As the triangulation approach calculates the causal effect (and its SE for testing significant deviation from null) by the ratio of the two IV based effect estimates, it is also known as ratio estimate or Wald estimate. It is important for the computation that both IV based effect estimates refer to the same allele of the IV. Furthermore, the same requirements as for the 2SLS apply. The SE of $\beta_{\hat{X}Y}$ has to be estimated via the delta-method which is based on a Taylor series expansion, and can be approximated as (20):

$$var\left(\beta_{\hat{X}Y}\right)=var\left(\frac{\beta_{ZY}}{\beta_{ZX}}\right)≅\frac{var\left(\beta_{ZY}\right)}{\beta_{ZX}^{2}}+\frac{\beta_{ZY}^{2}}{\beta_{ZX}^{4}}\;var\left(\beta_{ZX}\right)-2\;\frac{\beta_{ZY}}{\beta_{ZX}^{3}}\;cov\left(\beta_{ZY},\beta_{ZX}\right)$$

$SE\left(\beta_{\hat{X}Y}\right)=\sqrt{\;var\left(\beta_{\hat{X}Y}\right)}$, where $cov(\beta_{ZY},\beta_{ZX})$ is the covariance of the two effect estimates. This term will vanish if the effect estimates are obtained from distinct samples. That concise approximation can be easily implemented for significance testing in statistical software packages like R or STATA.

In contrast to the 2SLS which has to be performed using data of a single sample (one-sample MR), different sample sets can be used for conducting the triangulation: the effect estimates of the IV on exposure *X* and outcome *Y* can be obtained from genetic association studies with either disjunct or overlapping samples (two-sample MR). By this means, genetic associations revealed through large-scale GWAS meta-analyses can be used as $\beta_{ZX}$ and $\beta_{ZY}$. These association results are often publically available for a variety of traits.

The triangulation method can also be applied if the outcome *Y* is dichotomous, i.e., an indicator of a disease status. In this case, log-linear effects without interaction on *Y* and an approximately normal distribution of *X* are required. Causal effect estimates on the odds ratio (OR) scale can be calculated by performing a logistic regression analysis using the disease as outcome. This model was also applied in most GWAS. To estimate causal OR using triangulation, the rare disease assumption (i.e., prevalence <10%) has to be fulfilled. Alternatively, estimates of a causal risk ratio may be calculated using a log-linear model instead of a logistic regression (21). The SE of the $\beta_{\hat{X}Y}$ (i.e., the log causal OR) will be estimated by the same formula as applied in the case of a continuous outcome. An application of the ratio estimator is provided by the MR on cystatin c and cardiovascular disease (22).

### Control Function Estimator

Another method for estimating the causal effect on a dichotomous outcome is provided through the control function estimator (21) which is a two-step approach. In the first step, the exposure *X* is regressed on the instruments *Z*. The residuals of the regression correspond to the non-instrumented part of the exposure and may therefore correlate with a (unobserved) confounder *U* of the exposure-outcome association. In the second step, a logistic regression of the outcome *Y* on *X* is performed, adding the residuals of the first step as a covariate to the model. By adding the residuals of the first step into the model, the effects of *U* on *Y* will be controlled. Thus, the effect of *X* on *Y* of the second regression corresponds to the causal effect estimate. In case a linear regression is conducted in the second step (i.e., for a continuous outcome), the control function estimator is equivalent to the 2SLS estimator (21). This type of MR was conducted for assessing the causal effect of blood lipids on coronary heart disease (15).

### Assumptions of the Instrumental Variables

SNPs have several properties predisposing them for instruments of the exposure. The inherited alleles are not changed by a disease or trait and thus also do not change over time. The random inheritance of the SNP alleles makes the genotype distribution mostly independent from socio-economic and lifestyle factors (1, 23). Nevertheless, specific assumptions still need to be fulfilled to ensure the validity of the genetic variant as an instrument. There are three core assumptions for MR (24–26):

1. The genetic variant is associated with the exposure
2. The genetic variant is independent of the outcome given the exposure and all confounders (measured and unmeasured) of the exposure-outcome association
3. The genetic variant is independent of factors (measured and unmeasured) that confound the exposure-outcome relationship

The first condition is required because within the MR the (unconfounded) exposure will be estimated using the allele distribution of the IVs. This assumption can be easily tested, and is considered as fulfilled if the SNP-exposure association has an F-statistic >10 (21, 27).

The second assumption, which is also known as exclusion restriction, is equivalent to the condition that an IV does not have an effect on the outcome when the exposure remains fixed. In general, this assumption is hard to validate as there may be pleiotropic effects of SNPs or SNPs in linkage disequilibrium correlated with genes that have effects on the outcome independently of the exposure. Even without considering the linkage disequilibrium, using SNPs of the pleiotropic gene *GCKR* exemplarily as instruments for kidney function to assess a causal effect on blood pressure would result in an invalid IV as there are effects of *GCKR* on blood pressure likely that are independent of kidney function, e.g., by the known associations of *GCKR* with serum lipid levels. Another violation would occur if the sample consists of a population substructure with different allele distributions, and which is also associated with the outcome. In this case, the substructure would be a common cause of both SNP and outcome opening a pathway from SNP to outcome not mediated by the exposure. Several examples of different scenarios violating the exclusion restriction are provided in the work of Glymour et al. (24).

The third assumption is also hard to validate. Similar problems due to pleiotropy and population substructure as described in the exclusion restriction may occur but affecting confounders of the exposure-outcome relationship instead of the outcome directly. In an example of assessing causality of kidney function with heart disease, using *GCKR* as an instrument would violate the third assumption because these SNPs are also associated with blood pressure being a confounder of the association of kidney function and heart disease.

### Weak Instrument Bias

Until today, more than 50,000 SNP-trait associations were revealed by GWAS and are usually accessible through public repositories like the GWASCatalog (5). These SNPs can be considered as potential instruments for MR analyses. Because the majority of these SNPs explain only a small proportion (i.e. <1%) of the phenotypic variance, GWAS with sample sizes of more than 10,000 or 100,000 individuals were required to unravel these associations at the level of genome-wide significance. However, the small effect sizes of the SNPs on the exposure result in weak instruments when using smaller sample sizes (28). Weak instruments tend to lead estimated causal effects towards the observational association (27). The reason for this bias is originated in using finite sample sizes. Although the IVs are asymptotically independent of confounders, there might be still an association by chance in finite samples. Increasing the sample size or the strength of the instruments will reduce the weak instrument bias. To illustrate the origin and the effect of the bias, let $\beta_{UX}$ and $\beta_{UY}$ be the effects of the confounder *U* on the exposure and the outcome, respectively (Figure 1). Furthermore, let $\Delta_{U}$ be the (by chance) difference in* U* depending on the instrument *Z*. The estimated causal effect $\beta_{\hat{X}Y}$ can then be computed by the following sum of effects (27):

$\beta_{\hat{X}Y}=\beta_{causal}+\frac{\beta_{UY}\Delta_{U}}{\beta_{ZX}+\beta_{UX}\Delta_{U}}$, where as $\beta_{causal}$ is the true causal effect, and the mean($\Delta_{U}$) =0 because Z is an instrument (assumption 3). This leads the bias term towards zero with increasing sample size resulting in$\beta_{\hat{X}Y}=\beta_{causal}$. The estimated causal effect is also close to the true causal effect in case the effect of the IV on the exposure $\beta_{ZX}$ is relatively large compared to the by-chance difference in *U* on the exposure ($\beta_{UX}\Delta_{U}$). However, if $\beta_{ZX}$ is small compared to $\beta_{UX}\Delta_{U}$ (in case of a weak instrument), the estimated causal effect will be biased towards the ratio of the effect of the confounder on the outcome and the effect of the confounder on the exposure, i.e.,$\frac{\beta_{UY}}{\beta_{UX}}$.

### Multiple Instruments Approach

Using multiple valid instruments will help to address the weak instrument bias. Adding multiple uncorrelated (linkage equilibrium) SNPs into a 2SLS model can increase the statistical power but might also increase the relative bias if weak instruments are added (28).

Alternatively, an allele score can be generated from the instruments and included as a single variable in the association model. This allele score *Z* is calculated per individual as the weighted or unweighted sum of the number of risk or trait increasing alleles *Z_i_* of each SNP *i*, whereas the effect $\beta_{Z_{i}X}$ of each SNP on the exposure *X* is used as weight:$Z=\;\beta_{Z_{1}X}Z_{1}+\beta_{Z_{2}X}Z_{2}+\cdots +\beta_{Z_{k}X}Z_{k}$. In case of an unweighted score where all $\beta_{Z_{i}X}$ are set to 1, the allele score of an individual simplifies to the sum of its risk alleles. By using an allele score, the F-statistics increases because of the smaller degrees of freedom in the model. However, it has been shown that the unweighted score has lower power than adding multiple IVs into the 2SLS, but using an appropriately weighted allele score performs similarly. The causal effect is a little less biased when using a weighted allele score but might have a slightly lesser precision (and power) compared to the multiple IV 2SLS estimator. In general, effects obtained from external studies should be used as weights (28).

A third method for taking advantage of multiple IVs is to combine ratio estimates (triangulation) of single instruments using inverse variance weighting (29, 30). The method for combing the results is the same as used for meta-analyses, and is for example implemented in the R package *metafor*. Alternatively, the following simplification of this calculation can be used (31–33):

$$\beta_{\hat{X}Y}=\frac{\sum \beta_{ZX}\beta_{ZY}var{\left(\beta_{ZY}\right)}^{-1}}{\sum \beta_{ZX}^{2}var{\left(\beta_{ZY}\right)}^{-1}}$$

with its approximated $SE\left(\beta_{\hat{X}Y}\right)=\sqrt{\frac{1}{\sum \beta_{ZX}^{2}var{\left(\beta_{ZY}\right)}^{-1}}}$, where the sum runs over the SNP specific estimates. This method is implemented in the R package *gtx*.

However, it is crucial that the effects of all IVs used in the calculation are corresponding to the allele referring to the same effect direction on the exposure (e.g., the trait increasing allele). In theory, problems of missing data may occur especially when using multiple IVs. Nowadays well established methods for imputing missing genotypes based on the linkage disequilibrium structure of the human genome are available to circumvent this problem (34–36).

### Bias by Violation of the Assumptions 2 and 3

Importantly, valid instruments need to be included in the MR analyses. In case the assumptions are not fulfilled, different types of bias can occur leading to invalid causal effect estimates. Violation of the second assumption (the exclusion restriction) implies that there is at least a partial effect of the instrument on the outcome not mediated by the exposure, i.e. $\alpha_{ZY}\neq 0$ (Figure 1). Depending on the direction and strength of these pleiotropic effects, the causal effect will be over- or underestimated. As shown within the principle of triangulation, the estimated causal effect $\beta_{\hat{X}Y}$ is the sum of the true causal effect $\beta_{causal}$ and a bias term: $\beta_{\hat{X}Y}=\beta_{causal}+\frac{\alpha_{ZY}}{\beta_{ZX}}$ (26). The bias increases due to larger pleiotropy (larger absolute $\alpha_{ZY}$ in the nominator) or weaker instruments (smaller absolute $\beta_{ZX}$ in the denominator). Violation of assumption 3 leads to a bias similar to the weak instrument bias. In this case, the effect of the confounder *U* on exposure and outcome will not vary by chance but systematically because of the non-zero effect of the instrument *Z* on *U*. Thus, an increasing sample size will not remove the bias because mean($\Delta_{U}$) ≠ 0.

### InSIDE Condition and Egger MR

**Figure 2:**
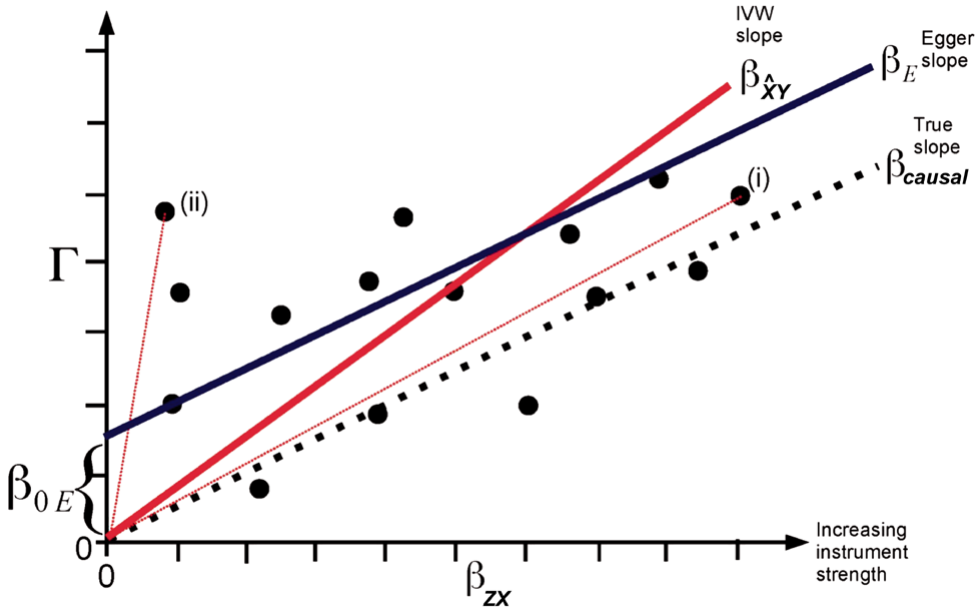
Plot of the SNP-outcome (Γ) on the y-axis vs. the SNP-exposure ($\beta_{ZX}$) regression coefficients of potential genetic instruments (i.e., SNPs) of a Mendelian randomization analysis on the x-axis. The true causal effect represented by the slope $\beta_{causal}$ is shown by a dotted line, the inverse variance weighted (IVW) causal estimate $\beta_{\hat{X}Y}$ by a red line, and the MR Egger regression estimate $\beta_{E}$ by a dark blue line. The total SNP-outcome effect Γ is proportional to $\beta_{ZX}$ for valid instruments. In case of invalid instruments but when the InSIDE assumption holds, stronger instruments are on average expected to be closer to the true causal effect (i) than weak instruments (ii). The intercept $\beta_{0E}$ represents the overall directional pleiotropy of the instruments. The figure was adapted from the publication of Bowden et al., Int J Epidemiol. 2015;44(2):512–525 (26) (Creative Commons CC BY license).

Pleiotropic effects $\alpha_{ZY}$ of each IV will also be included in the model when applying the multiple instruments approach. However, in this scenario it is possible to substitute the exclusion restriction by a weaker assumption as explained below. If the ratio estimates of multiple instruments are combined via 2SLS or inverse variance weighting, equation (1) will result in $\beta_{\hat{X}Y}=\beta_{causal}+\frac{\sum \beta_{ZX}\alpha_{ZY}var{\left(\beta_{ZY}\right)}^{-1}}{\sum \beta_{ZX}^{2}var{\left(\beta_{ZY}\right)}^{-1}}$ , where $\beta_{causal}$ equals the right side of (1) and $\frac{\sum \beta_{ZX}\alpha_{ZY}{var(\beta_{ZY})}^{-1}}{\sum {\beta_{ZX}}^{2}{var(\beta_{ZY})}^{-1}}$ is a bias depending on $\alpha_{ZY}$ and $\beta_{ZX}$. Thus, an unbiased causal effect will be obtained if the assumption 2 is true, i.e., all direct effects $\alpha_{ZY}$ of each IV on the outcome *Y* are zero. However, it will be sufficient for the bias term to equal zero if all pleiotropic effects $\alpha_{ZY}$ of all genetic IV cancel out. As shown below, this cancellation is sufficiently fulfilled if the correlation between direct genetic effects $\alpha_{ZY}$ on the outcome and their effects $\beta_{ZX}$ on the exposure *X* (i.e., the strength of the IV) is zero. This independence between the genetic effects $\alpha_{ZY}$ and $\beta_{ZX}$ is called InSIDE condition (Instrument Strength Independent of Direct Effect). If the InSIDE condition holds together with assumptions 1 and 3, an adaption of the Egger regression can be used to obtain a consistent causal estimate even for specific cases in which the exclusion restriction criteria is violated. The Egger regression for MR is an implementation of the meta-regression where the (total) SNP-outcome effect $\Gamma =\beta_{ZX}\beta_{causal}+\alpha_{ZY}$ for each SNP is regressed on the corresponding SNP-exposure effect $\beta_{ZX}$: $\Gamma \;∼\;\beta_{0E}+\beta_{E}\beta_{ZX}$ where the slope $\beta_{E}$ is the bias-reduced causal estimate (Figure 2). The principle behind this regression is that Γ is proportional to the strength of the instrument $\beta_{ZX}$ with the intercept $\beta_{0E}=0$ for valid instruments, whereas under the InSIDE condition (i.e., $\alpha_{ZY}$and $\beta_{ZX}$ are uncorrelated) stronger instruments are expected to have a relatively small bias and thus are on average closer to the true causal effect than weak instruments. As the slope of the Egger MR can be calculated by the least squares estimator $\beta_{E}=\frac{cov\left(\Gamma ,\beta_{ZX}\right)}{var\left(\beta_{ZX}\right)}=\beta_{causal}+\frac{cov\left(\beta_{ZX},\alpha_{ZY}\right)}{var\left(\beta_{ZX}\right)}$ , the bias term will be zero if $\alpha_{ZY}$ and $\beta_{ZX}$ are uncorrelated, which is the case under the InSIDE assumption. A non-zero intercept $\beta_{0E}$ indicates an overall directional pleiotropy of the IVs (26).

### Considering Statistical Power

The statistical power of an MR strongly depends on the proportion of variance of the exposure that is explained by the IV. The use of multiple IVs, either by direct inclusion or as an allele score in the model, may therefore increase the power as more variance of the exposure is explained. However, the validity of these instruments has to be ensured (37). Two-sample MR additionally provide a possibility to increase statistical power if published GWAS meta-analyses of both the exposure and the outcome are available. In this case, effect estimates based on large sample sizes of independent studies can be used to estimate the causal effect. Formulas for performing power calculations of MR using single instruments or allele scores are provided in the study of Burgess (37). Brion et al. (38) discusses the statistical power in case of single IV and continuous outcomes for 2SLS MR, and provide an online power calculator for both continuous and binary outcomes which is available at http://cnsgenomics.com/shiny/mRnd/. A tool for estimating statistical power of complex MR settings based on simulations is MR_predictor (39), whereas the PERL scripts required to run the estimator are available via GitHub.

### Measurement Unit of the Causal Effect Estimates

When conducting two-sample MR, the causal effect corresponds to the unit of the outcome on a per unit change of the exposure that was used in the respective genetic association study of the IV with the corresponding trait (32). Some GWAS were meta-analyzed using the sample-size weighted z-score method (40) and thus do not provide effect estimates that can be directly included in a two-sample MR. However, it is possible to estimate the effect $\hat{\beta }$ for each SNP in Hardy-Weinberg equilibrium using its minor allele frequency *MAF*, its (large) GWAS sample size *N*, and its z-statistics *z* (which can be calculated from the inverse of the standard normal distribution using the association p-value and the corresponding effect direction) through the formula (41): $\hat{\beta }\approx \;z\cdot \frac{\sigma }{\sqrt{N\cdot 2\cdot MAF\cdot \left(1-MAF\right)}}$, whereas the corresponding $SE\left(\hat{\beta }\right)=\frac{\hat{\beta }}{z}$. The SD *σ* of the trait can be set to 1 for standardizing the phenotype (i.e., the effect corresponds to a change of one SD of the trait unit). If the outcome is a binary trait, e.g., a disease with prevalence *p* in the sample, then $\sigma =\sqrt{p∙(1-p)}$.

## Discussion

MR provides a method for testing causality of different traits using cross-sectional data and genetics. Although large sample sizes are required to achieve sufficient statistical power for revealing causal effects, it is often possible to overcome this limitation by using the publically available genetic association results of large GWAS meta-analyses conducted during the last decade.

The statistical methods needed for conducting MR analyses are implemented in common statistical software frameworks. Additionally, the MRbase platform provides a possibility to conduct two-sample MR analyses both online and via the R package *TwoSampleMR*, including the methods discussed in this article (42). A detailed overview of different statistical methods for calculating MR is provided in the review of Burgess et al. (17).

However, it is important that the genetic associations that are used as instruments fulfil the MR assumptions to avoid calculation of biased or spurious causal estimates resulting in false causal inferences. Other than the required strong association of the genetic variant with the exposure, the remaining two assumptions are in general hard to validate.

This review emphasizes the bias that may occur by using invalid instruments, whereas the presented formulas should help estimating the magnitude and direction of this bias depending on the specific MR study that needs to be conducted. Using multiple instruments can help to test the violation of the MR assumptions which may occur due to pleiotropy and via SNPs in linkage disequilibrium (but not for a violation due to population stratification) (28), or to conduct sensitivity analyses (25). A strategy for assessing pleiotropy and population substructure specifically to MR analyses is discussed for example in the work of Lawler et al. (9). The Egger regression can be used as a multiple IV approach to relax the exclusion restriction criteria, and as a sensitivity analysis to test the robustness of the causal association (26). However, if the Egger MR-specific InSIDE assumption is violated, a biased causal estimate and an increased Type I error rate may occur (43). Thus, seeking for genetic variants that are valid IV should be performed as far as possible. Knowledge of the physiology or the biological pathways of the SNPs and their causal genes might be useful for selecting instruments.

The methods summarized in this review assume linear effects between exposure and outcome (or log-linear in case of a binary outcome) without effect modifications by the variables. Addressing these limitations is subject to future research. A method for successfully revealing non-linear causal effects was provided in an example for alcohol intake on cardiovascular traits, but this approach is restricted to additional assumptions and limitations (16). With respect to the presence of effect modifications, other statistical methods for conducting binary outcome MR like structural mean models or generalized method of moments make weaker assumptions but still not solve this issue completely (21). Finally, methods for automatically detecting invalid instruments (i.e., due to pleiotropy) are under development (44). Selection of valid instruments still remains a main challenge for automated causal inference.

## Author Contributions

AT designed and wrote the review.

## Conflict of Interest Statement

The author declares that the research was conducted in the absence of any commercial or financial relationships that could be construed as a potential conflict of interest.
